# Gut Microbial Communities Are Seasonally Variable in Warm-Climate Lizards Hibernating in the Winter Months

**DOI:** 10.3390/microorganisms12101974

**Published:** 2024-09-29

**Authors:** Xiaming Zhu, Neng Jiang, Tingye Mai, Shulin Wu, Yuntao Yao, Yu Du, Chixian Lin, Longhui Lin, Xiang Ji

**Affiliations:** 1 Herpetological Research Center, College of Life Sciences, Nanjing Normal University, Nanjing 210023, China; zhuxiaming@njnu.edu.cn (X.Z.); 221202136@njnu.edu.cn (N.J.); 2 Zhejiang Provincial Key Laboratory for Water Environment and Marine Biological Resources Protection, College of Life and Environmental Sciences, Wenzhou University, Wenzhou 325035, China; 3 Hainan Key Laboratory of Herpetological Research, College of Fisheries and Life Sciences, Hainan Tropical Ocean University, Sanya 572022, China; tymai@stu.hntou.edu.cn (T.M.); slwu@stu.hntou.edu.cn (S.W.); yaoyuntao@hntou.edu.cn (Y.Y.); yudu@hntou.edu.cn (Y.D.); chixianlin@hntou.edu.cn (C.L.); 4 Herpetological Research Center, College of Life and Environmental Sciences, Hangzhou Normal University, Hangzhou 311121, China

**Keywords:** gut microbiota, seasonal change, hibernation, warm climate, lizards

## Abstract

Hibernation is an energy-saving and adaptive strategy adopted by a diverse array of animals, rarely including warm-climate species, to survive in the harsh winter environment. Here, we collected large-intestinal microbial samples from two species of warm-climate lizards, one (the Reeves’ butterfly lizard *Leiolepis reevesii*) hibernating in the winter months and one (the many-lined sun skink *Eutropis multifasciata*) not doing so, in summer and winter to analyze and compare their microbiota using 16S rRNA gene amplicon sequencing technology. Gut microbiota were seasonally variable in *L. reevesii* but not in *E. multifasciata*. The decreased Firmicutes/Bacteroidetes ratio and increased relative abundance of Verrucomicrobia in hibernating butterfly lizards in a state of long-term fasting should help them live through the winter months, as bacteria of the phyla Bacteroidetes and Verrucomicrobia can use host-derived mucin glycans in the absence of dietary substrates. Facultative plant feeding by omnivorous butterfly lizards resulted in a significant increase in the relative abundance of bacteria of the phylum Firmicutes (e.g., Lachnospiraceae) with the ability to degrade plant fibers. This study not only validates the role of gut microbiota in dietary adaptation in lizards but also shows that gut microbial communities are seasonally variable in warm-climate lizards hibernating in the winter months.

## 1. Introduction

The coevolution of a host and its gut microbiota is determined by numerous internal and external factors [1,2,3]. Diet as an external factor drives the convergent evolution of gut microbiomes [4,5]. For example, the specialized bamboo diet drives the giant panda *Ailuropoda melanoleuca* and the red panda *Ailurus styani* to harbor more bacteria related to starch and sucrose metabolism and vitamin B12 biosynthesis than carnivorous counterparts [4]. Diet primarily selects for functional guilds of bacteria (e.g., cellulolytic consortia), while host phylogeny mainly determines the relative abundance of bacteria taxa among vertebrate clades [6]. Seasonal changes in internal and external factors also affect the gut microbial composition and function [7,8,9,10]. For example, freely grazing yaks (*Bos grunniens*) shift the *Ruminococcaceae_UCG-005* enterotype in the warm season (high protein and low fiber diets) to the *Akkermansia* and uncultured *Eubacterium WCHB1-41* enterotype in the cold season (low protein and high fiber diets) [9]. In the plateau pika *Ochotona curzoniae*, temporal changes in ambient temperature, precipitation, and nutritional composition (crude protein, crude fiber, and polysaccharide) from vegetation drive seasonal dynamics in gut microbiota, and nutritional composition has a higher contribution to gut microbial variation than climatic factors [8].

As an energy-saving and adaptive strategy adopted by a diverse array of animals to survive in the harsh winter environment, hibernation significantly alters the composition of gut microbiota [11,12,13,14,15,16]. For example, bacteria of the phyla Bacteroidetes and Verrucomicrobia are enriched in winter, and bacteria of the phylum Firmicutes are enriched in the active season in small mammals such as the thirteen-lined ground squirrel *Ictidomys tridecemlineatus* [11,12], the arctic ground squirrel *Urocitellus parryii* [15], and the greater horseshoe bat *Rhinolophus ferrumequinum* [16] hibernating in winter, and the relative abundance of the phylum Verrucomicrobia decreases in hibernating brown bears (*Ursus arctos*) [13]. Hibernation also significantly alters the composition of gut microbiota in ectothermic animals. In the Chinese alligator *Alligator sinensis*, the relative abundance of bacteria (e.g., species of the genus *Bacteroides*) of the phylum Bacteroidetes increases in winter because these bacteria can use host-derived mucin glycans and are therefore beneficial for the hibernating host in a state of long-term fasting [17]. In the Mongolian toad *Strauchbufo raddei*, the phylum Proteobacteria predominates in hibernating individuals, and the increased relative abundances of bacterial genera (e.g., *Pseudomonas*, *Vibrio*, and *Ralstonia*) of the phylum are positively correlated with the metabolites associated with fatty acid metabolism and biosynthesis of unsaturated fatty acids [18]. The bacteria and metabolites enriched in hibernating Mongolian toads are beneficial for them to live through the winter months [18].

Hibernation rarely occurs in warm-climate regions where temperatures are relatively high and stable. Nevertheless, several lines of evidence from a diverse array of animal taxa, including mammals [19,20] and reptiles [21], prove that hibernation does occur in some warm-climate animals. For example, Malagasy lemurs (*Cheirogaleus medius*) hibernate in tree holes for seven months every year even when ambient temperatures are above 30 °C [19], and clouded monitor lizards (*Varanus nebulosus*) enter an inactive period when the daily mean temperature is below 22 °C [21].

Here, we focused on two sympatric species of warm-climate lizards, the Reeves’ butterfly lizard *Leiolepis reevesii* and the many-lined sun skink *Eutropis multifasciata*. *Leiolepis reevesii* is a medium-sized (up to 166 mm snout-vent length, SVL) omnivorous agamid lizard hibernating from late November to early March at a mean nest temperature of ~27 °C, with females producing a single clutch of 2–8 eggs per year from April to July [22]. *Eutropis multifasciata* is a medium-sized (up to 117 mm SVL) insectivorous sincid lizard not hibernating in the winter months, with females giving birth to 2–7 young per year from April to June [23]. Specifically, we collected large-intestinal content samples from these two species in summer and winter, analyzed and compared the seasonality of gut microbial composition and abundance using 16S rRNA gene amplicon sequencing technology, and tested the following two hypotheses: First, gut microbial diversity and composition should differ between *L. reevesii* and *E. multifasciata* because of their difference in food habits. Second, gut microbial communities should be seasonally more variable in *L. reevesii* than in *E. multifasciata* because of the role of hibernation in altering the composition of gut microbiota.

## 2. Materials and Methods

### 2.1. Sample Collection

We collected 17 adult *E. multifasciata* (four females and four males in December 2020, and five females and four males in August 2021) and 22 adult *L. reevesii* (four females and seven males in December 2018, and five females and six males in June 2019) from Ledong (18°29′–18°31′ N, 108°47′–108°57′ E), Hainan Island, South China. We dissected lizards on the day they were captured for collection of large-intestine contents, which were individually labeled and then stored at −80 °C for later DNA extraction.

Our work was carried out in compliance with current laws on animal welfare and research in China and approved by the Animal Research Ethical Committee of Hainan Tropical Ocean University (Approval number: IACUC-20180501).

### 2.2. DNA Extraction and Sequencing

We used the CTAB/SDS method to extract microbial DNA from large-intestinal samples, assessing concentration and purity with Nanodrop (Thermo Fisher Scientific, Waltham, MA, USA) and 1.2% agarose gels. The V3–V4 region of the bacterial 16S rRNA was amplified by PCR using the forward primer 338F (5′-ACTCCTACGGGAGGCAGCA-3′) and the reverse primer 806R (5′-GGACTACHVGGGTWTCTAAT-3′). The PCR reaction mixture contained 5 μL of buffer (5×), 2 μL of dNTPs (2.5 mM), 1 μL of each primer (10 uM), 0.25 μL of Fast pfu DNA Polymerase, 1 μL of DNA template, and 14.75 μL of ddH_2_O. The PCR thermal cycling conditions were as follows: pre-denaturation at 98 °C for 5 min, denaturation at 98 °C for 30 s, annealing at 53 °C for 30 s, extension at 72 °C for 45 s, reacting for 25 cycles, and finally extending at 72 °C for 5 min. PCR amplicons were purified with Vazyme VAHTSTM DNA Clean Beads (Vazyme, Nanjing, China) and quantified using the Quant-iT PicoGreen dsDNA Assay Kit (Invitrogen, Carlsbad, CA, USA). All samples were mixed with an equal molar amount from the purified PCR product of each sample, and a library was prepared using the TruSeq Nano DNA LT Library Prep Kit (Illumina, Sangon Biotech Co., Ltd., Shanghai, China). Purified amplicon DNA libraries were sequenced using a MiSeq Reagent Kit V3 (Illumina, Sangon Biotech Co., Ltd., Shanghai, China) on an Illumina MiSeq platform (San Diego, CA, USA) in accordance with Frasergen Bioinformatics (Wuhan, China).

### 2.3. Bioinformatics

We used the Quantitative Insights Into Microbial Ecology 2 (QIIME2) 2020.8 (https://qiime2.org/; accessed on 25 November 2023) to process and analyze raw sequences, the CUTADAPT plugin to trim primers, and the DADA2 plugin to filter reads based on quality, merge paired reads, remove chimeras, and assign reads to amplicon sequence variants (ASVs) [24,25]. We finally obtained 2,359,766 (60,506 ± 2019) high-quality reads from the aforementioned 39 lizards: 17 *E. multifasciata* and 22 *L. reevesii*. The obtained sequences were searched against the Greengenes 13_8_99% OTU reference database for taxonomic assignment [26] and reads identified as the sequences from archaea, chloroplast, and mitochondria were removed. A phylogenetic tree was constructed using the MAFFT 7.480 alignment algorithm and the FastTree 2.1.10 maximum likelihood estimation [27,28]. To avoid sample deviations, ASVs with more than 20 reads and present in more than two samples were retained. To standardize the ASV abundance information, the sequencing depth was rarefied at 29,217 according to the 95% of the lowest number of sequences of one sample. Alpha and beta diversity metric values were calculated using the “core-metrics-phylogenetic” pipeline through the QIIME2 q2-diversity plugin. A total of 2164 ASVs were obtained after filter and normalization. Rarefaction curves of estimated ASVs and Shannon indexes indicated that sequence depths were sufficient to describe of the bacterial community (Figure S1).

### 2.4. Statistical Analysis

We used four alpha diversity indexes to assess the community richness (observed ASVs, Faith’s phylogenetic diversity), diversity (Shannon), and evenness (Pielou’s evenness). Two-way ANOVA was used to examine the effects of host species (*E. multifasciata* versus *L. reevesii*), season (summer versus winter), and their interactions on alpha diversity indexes. We used Jaccard and unweighted UniFrac distances to assess community membership, taking the presence and absence of microbial lineages into account; we used Bray–Curtis and weighted UniFrac distances to assess community structure, taking the relative abundances of microbial lineages into account. Jaccard and Bray–Curtis distances belong to non-phylogenetic-based distances, and unweighted and weighted UniFarc distances belong to phylogenetic-based distances. The principal coordinates analysis (PCoA) ordinations were used to visualize structural variations in microbial communities. Permutational multivariate analysis of variance (PERMANOVA, permutation = 999) was performed using the adonis function (vegan package in R 4.3.2) [29] to test the effects of host species, season, and their interaction on these two distance matrixes. A beta dispersion test was performed using the betadisper function (vegan package) to test the homogeneity of dispersion between the two species and between the two seasons. Descriptive statistics were expressed as mean ± standard error (SE) and the significance level was set at *p* = 0.05.

Linear discriminant analysis (*LDA*) effect size (LEfSe) [30] was performed to compare the relative abundance of taxa (at the phylum and family levels) with total relative abundances > 1% among the different host species × season combinations. Biomarker species in each of the four (2 host species × 2 seasons) combinations were detected by the Kruskal–Wallis test and linear discriminant analysis (*LDA* > 3 and *p* < 0.05). Mann–Whitney *U* test was performed to compare the relative abundance of taxa between summer and winter in each lizard species. At the ASV level, the differentially abundant ASVs were detected using the metagenomeSeq package [31] and visualized as Manhattan plots.

To obtain the best discriminant performance of the taxa between summer and winter, a random forest model was performed at the family and ASV levels using randomForest (ntree = 10,000) package 4.7-1.1 [32]. The cross-validation error curve of the 10-fold cross-validation was performed by the rfcv function (100 iterations) to identify the number of Biomarker taxa.

Co-occurrence networks were constructed based on Spearman’s pairwise correlations of ASVs using the psych package [33]. To reduce the complexity, ASVs with total relative abundances > 0.1% and present in at least three samples in each group were retained. ASV pairs with a significant correlation (|*ρ*| > 0.7, *p* < 0.01) were extracted and visualized using the igraph package [34] and the Gephi 0.9.7 software [35].

## 3. Results

### 3.1. Microbial Community Diversity

**Figure 1:**
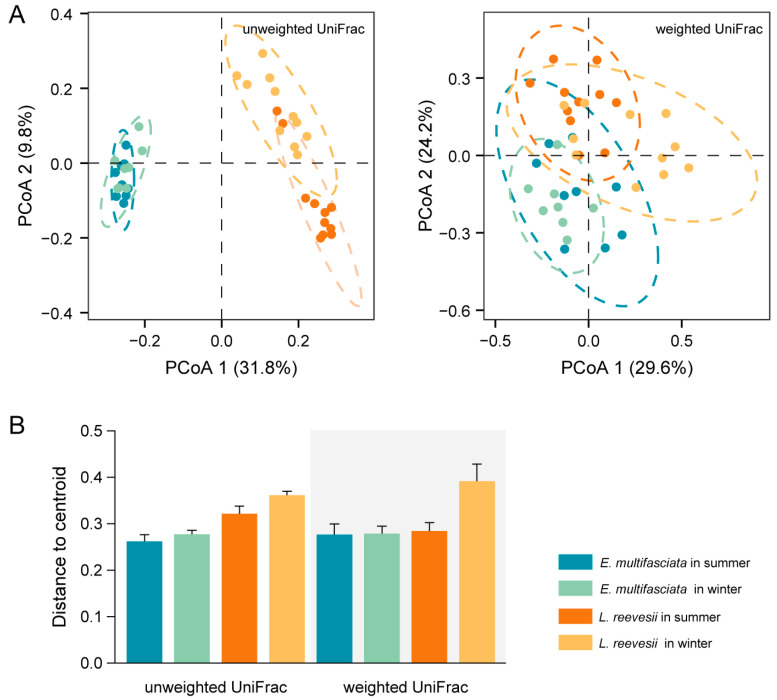
Results of principal coordinates analysis (PCoA) on gut microbiota (**A**) and mean values (+SE) for distance to centroid (**B**) in a combination of two host species (*E. multifasciata* and *L. reevesii*) × two seasons (summer and winter). Each color in the figure represents a species × season combination (See the explanation on the right side of Plot B).

All four alpha diversity indexes were significantly greater in summer than in winter, three (Observed ASVs, Shannon, and Evenness) of the four indexes were significantly greater in *E. multifasciata* than in *L. reevesii*, and the season × species interaction was a significant source of variation in two (Shannon and Evenness) of the four indexes (Table 1). The microbial community membership and structure differed between the two species and between the two seasons, and the season × species interaction was a significant source of variation in these two variables (Figure 1A and Figure S2, Table S1). The microbial community membership and structure differed between summer and winter in *L. reevesii* but not in *E. multifasciata* (Table S1). Unweighted and weighted UniFrac distances to centroid revealed the following (Figure 1B): First, gut microbiota were more dispersed in *L. reevesii* than in *E. multifasciata* (unweighted: *F*_1, 35_ = 31.29, *p* < 0.01; weighted: *F*_1, 35_ = 5.22, *p* = 0.03). Second, gut microbiota were more dispersed in winter than in summer (unweighted: *F*_1, 35_ = 4.62, *p* < 0.05; weighted: *F*_1, 35_ = 4.26, *p* < 0.05). Third, the host species × season interaction was not a significant source of variation in the degree of dispersion (unweighted: *F*_1, 35_ = 0.92, *p* = 0.34; weighted: *F*_1, 35_ = 3.94, *p* = 0.06). Jaccard and Bray–Curtis distances revealed the following: First, gut microbiota were more dispersed in *L. reevesii* than in *E. multifasciata* (Jaccard: *F*_1, 35_ = 33.61, *p* < 0.01; Bray–Curtis: *F*_1, 35_ = 14.02, *p* < 0.01). Second, season was not a significant source of variation in the degree of dispersion (Jaccard: *F*_1, 35_ = 2.81, *p* = 0.10; Bray–Curtis: *F*_1, 35_ = 2.20, *p* = 0.15), nor was the host species × season interaction (Jaccard: *F*_1, 35_ = 0.96, *p* = 0.33; Bray–Curtis: *F*_1, 35_ = 1.21, *p* = 0.28).

### 3.2. Microbial Community Composition

**Figure 2:**
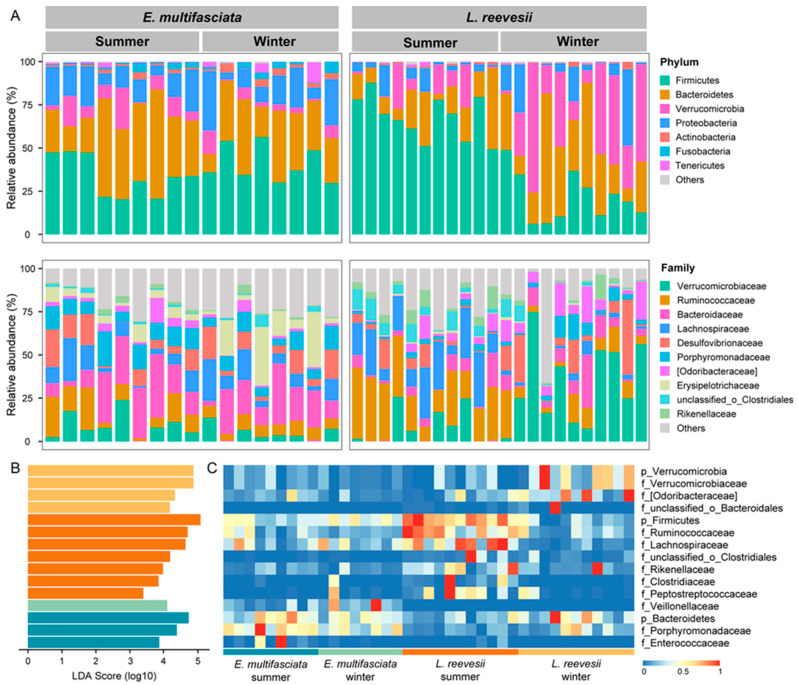
The relative abundance of gut microbiota at the phylum and family levels in a combination of two host species × two seasons (**A**), the three phyla and 12 families of gut microbiota identified by LEfSe that differed significantly in relative abundance among the four combinations (**B**), and heatmap showing the relative abundance of each significantly differentially abundant phylum or family (**C**).

**Figure 3:**
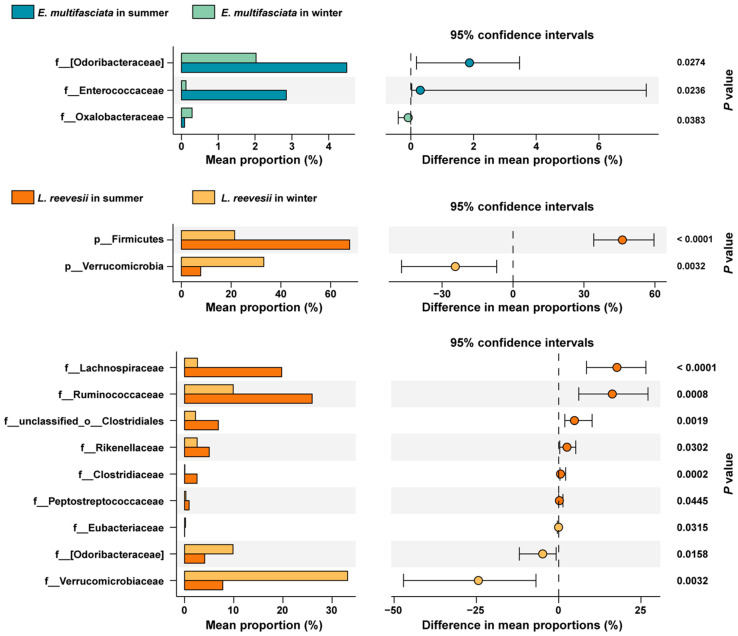
The bacterial phyla and families that differed significantly in relative abundance between summer and winter in each lizard species.

Firmicutes (37.0 ± 2.8% in *E. multifasciata* and 44.6 ± 5.8% in *L. reevesii*), Bacteroidetes (33.5 ± 3.5% in *E. multifasciata* and 25.1 ± 3.9% in *L. reevesii*), Verrucomicrobia (7.2 ± 1.5% in *E. multifasciata* and 20.5 ± 4.7% in *L. reevesii*), and Proteobacteria (16.1 ± 2.1% in *E. multifasciata* and 8.4 ± 2.4% in *L. reevesii*) were the top four dominant phyla in both species (Figure 2A). The LEfSe results showed that three of the top four dominant phyla differed among the four host species × season combinations in terms of relative abundance. Specifically, bacteria of the phylum Bacteroidetes were enriched in *E. multifasciata* in summer (*LDA* = 4.73, *p* = 0.04), bacteria of the phylum Firmicutes were enriched in *L. reevesii* in summer (*LDA* = 5.07, *p* < 0.01), and bacteria of the phylum Verrucomicrobia were enriched in *L. reevesii* in winter (*LDA* = 5.22, *p* < 0.01) (Figure 2B,C). In *L. reevesii*, bacteria of the phylum Firmicutes were more abundant in summer than in winter, and bacteria of the phylum Verrucomicrobia were more abundant in winter than in summer (Mann–Whitney test, both *p* < 0.01) (Figure 3). The Firmicutes/Bacteroidetes ratio decreased from 6.60 in summer to 1.06 in winter in *L. reevesii*, and the difference was significant (*U* = 112, *df* = 1, *p* < 0.01); the ratio did not differ between the two seasons in *E. multifasciata* (*U* = 49, *df* = 1, *p* = 0.24).

Twelve microbial families differed among the four host species × season combinations in terms of relative abundance (Figure 2B). Bacteria of the families Enterococcaceae (*LDA* = 3.86, *p* < 0.01) and Porphyromonadaceae (*LDA* = 4.38, *p* < 0.01) were enriched in *E. multifasciata* in summer. Bacteria of the family Veillonellaceae were enriched in *E. multifasciata* in winter (*LDA* = 4.10, *p* < 0.01). Bacteria of the families Clostridiaceae (*LDA* = 3.84, *p* < 0.01), Lachnospiraceae (*LDA* = 4.63, *p* < 0.01), Peptostreptococcaceae (*LDA* = 3.39, *p* < 0.01), Rikenellaceae (*LDA* = 3.97, *p* = 0.02), Ruminococcaceae (*LDA* = 4.70, *p* < 0.01), and unclassified_o_Clostridiales (*LDA* = 4.18, *p* < 0.01) were enriched in *L. reevesii* in summer. Bacteria of the families Odoribacteraceae (*LDA* = 4.32, *p* = 0.01), Verrucomicrobiaceae (*LDA* = 4.87, *p* < 0.01), and unclassified_o_Bacteroidales (*LDA* = 4.17, *p* = 0.01) were enriched in *L. reevesii* in winter. Bacteria of the families Odoribacteraceae and Enterococcaceae were more abundant in summer than in winter and bacteria of the family Oxalobacteraceae were more abundant in winter than in summer in *E. multifasciata* (Mann–Whitney test, all *p* < 0.04) (Figure 3). Bacteria of the families Lachnospiraceae, Ruminococcaceae, unclassified_o_Clostridiales, Rikenellaceae, Clostridiaceae, and Peptostreptococcaceae were more abundant in summer than in winter, and bacteria of the families Verrucomicrobiaceae, Odoribacteraceae, and Eubacteriaceae were more abundant in winter than in summer in *L. reevesii* (Figure 3).

**Figure 4:**
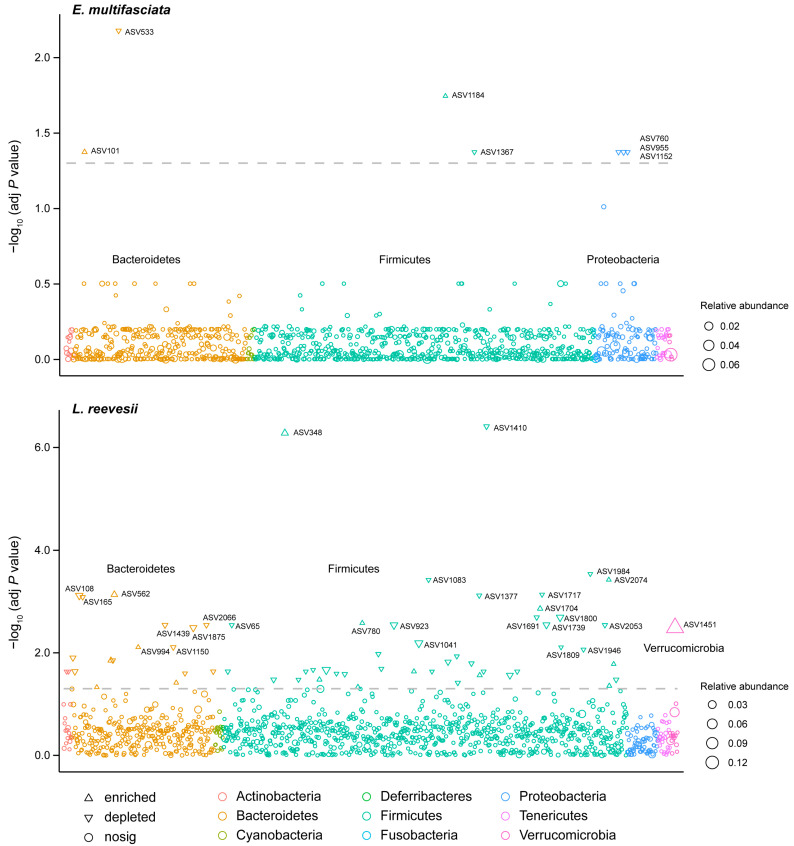
Manhattan plots showing the ASVs that differed significantly in relative abundance between summer and winter. Each triangle or dot represents a single ASV colored by phylum. The dashed line shows the significance threshold (adjusted *p* < 0.05).

The number of ASVs differing between summer and winter in terms of relative abundance was seven in *E. multifasciata* and 63 in *L. reevesii* (Figure 4, Table S2). Of the seven ASVs in *E. multifasciata*, two [ASV101 of the family Bacteroidaceae (phylum Bacteroidetes) and ASV1184 of the family Bacillaceae (phylum Firmicutes)] were enriched in winter, and five [ASV533 of the family Bacteroidaceae (phylum Bacteroidetes), ASV760, ASV955, and ASV1152 of the family Enterobacteriaceae (phylum Proteobacteria), and ASV1367 of the family Lachnospiraceae (phylum Firmicutes) were enriched in summer. Of the 63 ASVs in *L. reevesii*, 16 of the phyla Bacteroidetes, Firmicutes, and Verrucomicrobia were enriched in winter, and the remaining 47 of the phyla Actinobacteria, Bacteroidetes, and Firmicutes were enriched in summer. ASV348 and ASV1410 of the family Ruminococcaceae (phylum Firmicutes) and ASV1451 of the family Verrucomicrobiaceae (phylum Verrucomicrobia) were most typical in *L. reevesii*, with ASV348 enriched in winter, ASV1410 in summer, and ASV1451 in winter.

### 3.3. Biomarker Taxa for L. reevesii in Summer and Winter

**Figure 5:**
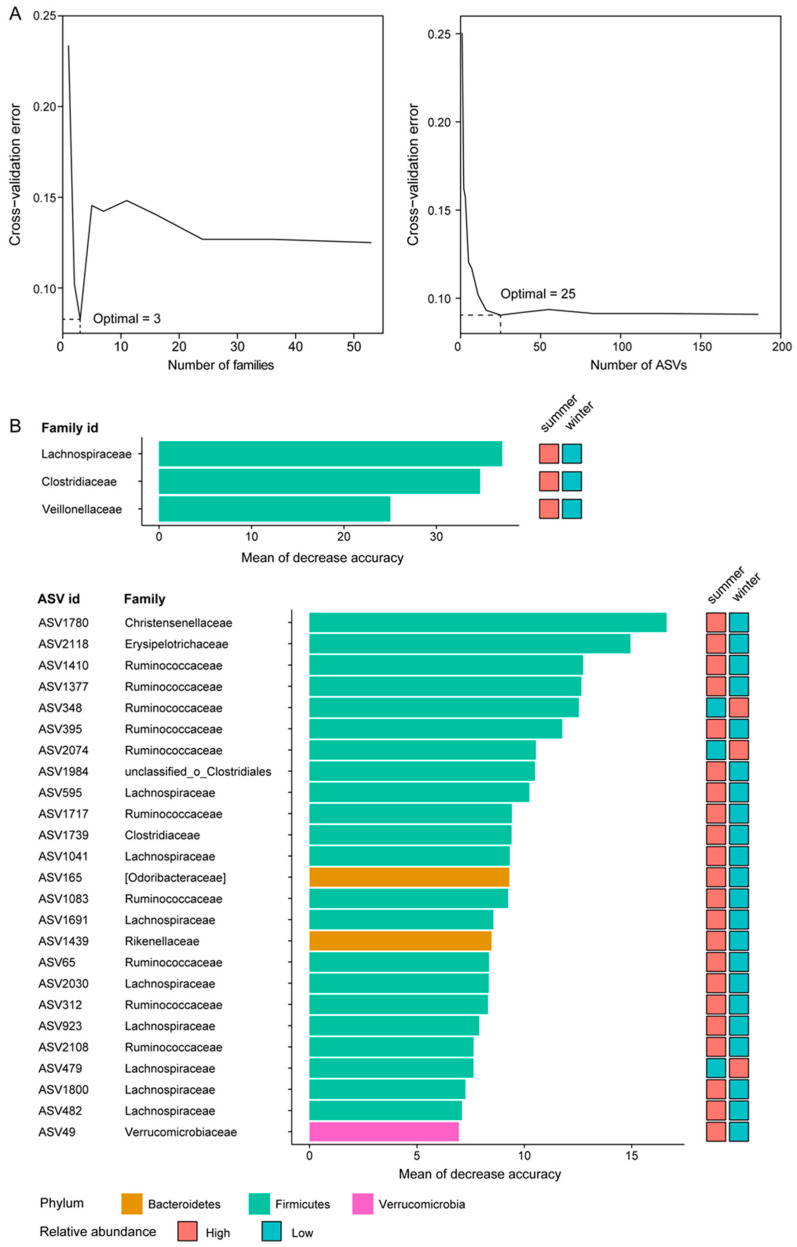
Random forest model detects biomarker taxa that are most associated with summer and winter individuals of *L. reevesii*. (**A**) 10-fold cross-validation error as a function of the number of family or ASV used to differentiate gut microbiota between summer and winter (**A**), and the top three families and 25 ASVs identified by a random forest model (**B**). Biomarker taxa are ranked in descending order of their importance to the accuracy of the model.

The random forest model showed that the accuracy of microbial classification between individuals of *L. reevesii* in summer and winter was 81.9% at the family level and 90.9% at the ASV level. The minimum cross-validation error was achieved by using the three most relevant families (Lachnospiraceae, Clostridiaceae, and Veillonellaceae) and 25 ASVs (Figure 5A), which were considered biomarker taxa. These three families and 22 of the 25 ASVs all belonged to the phylum Firmicutes (Figure 5B). Of the 22 ASVs, 10 belonged to the family Ruminococcaceae and eight belonged to the family Lachnospiraceae. Of these 28 biomarker taxa, 25 (three families and 22 ASVs of the phylum Firmicutes) showed higher relative abundance in summer, and the remaining three (ASV348, ASV479, and ASV2074) showed higher relative abundance in winter (Figure 5). Based on the Mann–Whitney test and metagenomeSeq, two families (Lachnospiraceae and Clostridiaceae) and 19 ASVs (other than ASV49, ASV312, ASV395, ASV479, ASV2030, and ASV2118) differed significantly in relative abundance between individuals of *L. reevesii* in summer and winter (Figure 3 and Figure 4).

### 3.4. Co-Occurrence Networks

**Figure 6:**
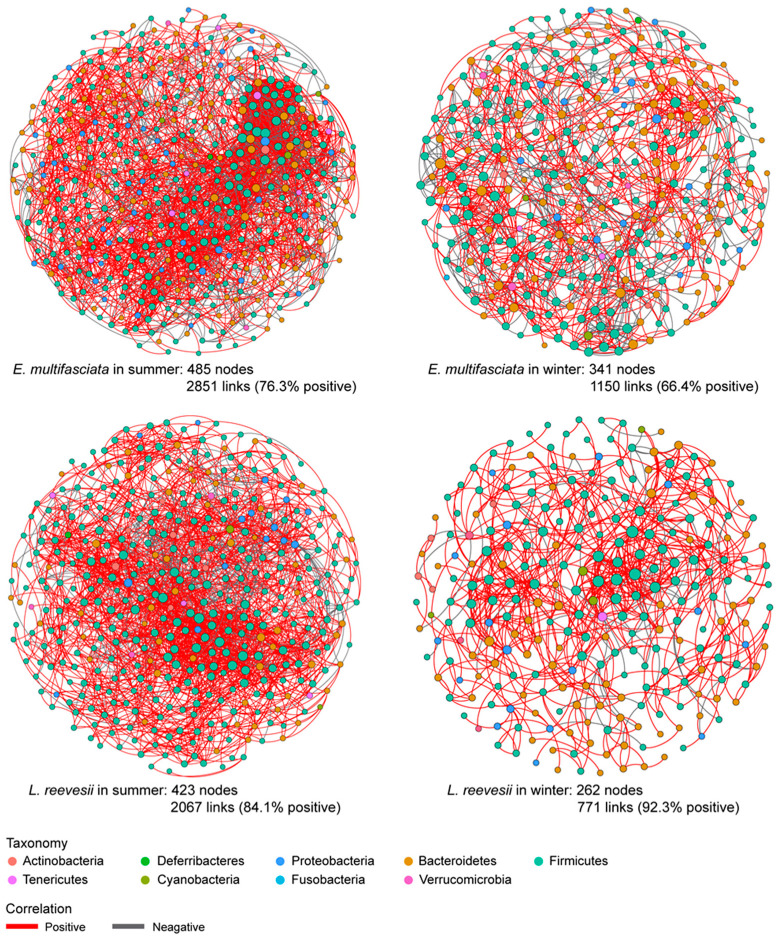
Co-occurrence networks of ASVs with total relative abundances > 0.1% and present in at least three samples in each of the four host species × season combinations. Nodes represent ASVs, which are colored by phylum and sized by relative abundance. Links between nodes represent a significant correlation (|*ρ*| > 0.7, *p* < 0.01) between two ASVs, which are colored by direction (red: positive; grey: negative).

Gut microbial co-occurrence networks were more complex in summer than in winter in both species. In summer, the average connectivity degree was 11.8 in *E. multifasciata* and 9.8 in *L. reevesii*, and in winter, it was 6.7 in *E. multifasciata* and 5.9 in *L. reevesii*. Similarly, the number of connections (links) in the networks was greater in summer (2851 in *E. multifasciata* and 2067 in *L. reevesii*) than in winter (1150 in *E. multifasciata* and 771 in *L. reevesii*) (Figure 6). Most links (>84%) in the co-occurrence network were positively correlated in *L. reevesii*, whereas negatively correlated links increased over 23% in *E. multifasciata* (Figure 6). Firmicutes and Bacteroidetes were the top two dominant phyla within the co-occurrence networks in both species. Seasonal shifts in the taxonomic composition of the networks were evident in *L. reevesii*, as revealed by the fact that bacteria of the phylum Bacteroidetes accounted for 14.7% in summer and 30.2% in winter.

## 4. Discussion

Alpha diversity of gut microbiota was higher in *E. multifasciata* than in *L. reevesii*, and the two species were separated in summer and winter in terms of beta diversity of gut microbiota based on four beta diversity matrixes (Jaccard, Bray–Curtis, and unweighted and weighted UniFrac distances). These findings suggest that, as in insects [36], sea turtles [37], and woodrats [2], the gut microbiota are species-specific in warm-climate lizards. Alpha diversity of gut microbiota was higher in summer than in winter in both species, with the lowest community diversity and evenness found in *L. reevesii* in winter. The finding that the alpha diversity of gut microbiota is higher in summer than in winter is consistent with the studies on *S. raddei* [18] and *O. curzoniae* [38]. Higher bacterial diversity of gut microbiota in summer may be due to more diverse and easily accessible food items, higher temperatures, and more frequent precipitation in the season. For example, the Shannon index of gut microbiota is positively associated with cumulative rainfall in the Ethiopian gelada *Theropithecus gelada* [7]. Hainan has only two seasons throughout the year: rainy season (from May to October) and dry season (from November to April) [39]. Higher alpha diversity of gut microbiota in summer in the two lizard species could be at least partially related to more precipitation in the season. Seasonality in beta diversity of gut microbiota has been reported for mammals such as the furry-eared dwarf lemur *Cheirogaleus crossleyi* [40] and the greater horseshoe bat *Rhinolophus ferrumequinum* [16] hibernating in winter. Here, seasonality in microbial community membership and structure was detected only in *L. reevesii* also hibernating in the winter months.

Two phyla, nine families, and 63 ASVs differed in relative abundance between summer and winter in *L. reevesii*, whereas only three low-abundance families and seven ASVs differed in relative abundance between summer and winter in *E. multifasciata*. This inter-specific difference provides an inference that gut microbial communities are seasonally less variable in warm-climate lizards such as *E. multifasciata* not hibernating in the winter months. Less pronounced seasonal variation in gut microbial communities has been reported for several species of warm-climate animals not hibernating in winter, including two spiny lizards of the genus *Sceloporus* (*S. grammicus* and *S. spinosus*) [41] and the François’ langur *Trachypithecus francoisi* [42]. In *T. francoisi*, the absence of significant seasonality in gut microbial diversity and relative abundance of high-abundance taxa primarily results from the stability of host activity rhythm and energy metabolism [42].

Bacteria of the phylum Firmicutes and families Clostridiaceae, Lachnospiraceae, Ruminococcaceae, Peptostreptococcaceae, and unclassified_o_Clostridiales of the phylum were significantly enriched in *L. reevesii* in summer. It is worth noting that bacteria of the families Ruminococcaceae and Lachnospiraceae are dedicated plant fiber degraders with the ability to ferment polysaccharides to generate short-chain fatty acids (SCFAs, e.g., butyrate and acetate) [43,44]. These bacterial taxa are therefore particularly critical for herbivorous or omnivorous animals [45,46,47,48]. For example, bacteria of the families Ruminococaceae and Lachnospiraceae account for 3–30% of rectal and fecal bacterial operational taxonomic units (OTUs) in the green turtle *Chelonia mydas* [45], and SCFAs are the main product of fermentation of plant material in the large intestine of this omnivorous sea turtle [49]. Relative abundance of bacteria of the family Lachnospiraceae decreases in green turtles fed with 25% plant and 75% seafood feedstuffs compared with those fed with 50% or higher proportions of plant feedstuffs [50]. The Italian wall lizard *Podarcis siculus* provides an example where the relative abundance of bacteria of the family Peptostreptococcaceae is higher in insectivorous populations than in omnivorous populations [51]. Our unpublished DNA metabarcoding data reveal that *E. multifasciata* is an insectivorous species and that *L. reevesii* is an omnivorous species eating not only arthropods but also fresh leaves of the orders Poales, Caryophyllales, Gentianales, and Brassicales. Higher relative abundance of bacteria of the phylum Firmicutes helps *L. reevesii* degrade plant fibers, further proving the contribution of the microbial community to dietary adaptation in lizards.

Hibernation significantly altered the gut microbial composition in *L. reevesii*. Firmicutes bacteria are more efficient in dietary caloric absorption than Bacteroidetes bacteria [52,53,54], and SCFAs produced by Firmicutes bacteria can be directly absorbed by the host as an energy source to promote mass gain [55]. Warming reduces the relative abundances of Firmicutes bacteria in many vertebrates [56,57], including the western fence lizard *Sceloporus occidentalis* [58]. However, the correlation between the relative abundances of Firmicutes bacteria and temperature also can be positive [59,60] or nonsignificant [61] in lizards. Bacteria of the phylum Bacteroidetes can use host-derived mucin glycans in the absence of dietary substrates [11,62]. A high Firmicutes/Bacteroidetes ratio explains weight gain in birds [63] and mammals [64]. In an earlier study on *L. reevesii*, the Firmicutes/Bacteroidetes ratio was reported to be greater in individuals acclimated to higher temperatures [59]. The Firmicutes/Bacteroidetes ratio is also related to the alteration in host status from the active to the fasting phase of hibernation [12,13,15,62]. Several lines of evidence from the brown frog *Rana dybowskii* [65], *I. tridecemlineatus* [12], and *U. arctos* [13] consistently show that the decreased relative abundance of Firmicutes bacteria and the increased relative abundance of Bacteroidetes bacteria help hosts adapt to long-term fasting during hibernation. In *R. dybowskii*, for example, the Firmicutes/Bacteroidetes ratio decreases from 4.23 in summer to 0.63 in winter [65]. Here, we found that the Firmicutes/Bacteroidetes ratio reduced in warm-climate lizards hibernating in the winter months, as revealed by the fact that the ratio did not differ between the two seasons in *E. multifasciata* but decreased from 6.60 in summer to 1.06 in winter in *L. reevesii*.

Hibernation decreased the relative abundance of bacteria (e.g., those of the families Ruminococcaceae and Lachnospiracea) using dietary glycans and increased the relative abundance of bacteria (e.g., those of the genus *Bacteroides*) [62] shifting their foraging targets between dietary glycans and host-derived mucin glycans. Hibernation also increased the relative abundance of bacteria (e.g., those of genus *Akkermansia*) degrading host-derived mucin glycans [11,12,13,15,17,65,66]. Bacteria of the genus *Akkermansia* are dedicated degraders of host-derived mucin glycans that can grow with mucin as the sole source of carbon and nitrogen [66,67]. In this study, we found that, as in *I. tridecemlineatus* during hibernation [12], bacteria of the family Verrucomicrobiaceae (with relative abundance mainly determined by the genus *Akkermansia*) were enriched in hibernating *L. reevesii*. Previous studies on the Syrian hamster *Mesocricetus auratus* [14] and *U. parryii* [15] showed that fasting, instead of low temperature *per se*, was the major factor determining the composition and metabolic output of gut microbiota during hibernation. The relative abundance of *Akkermansia* bacteria was higher and the concentration of total SCFAs was lower in active Syrian hamsters fasted for 96 h than in their active counterparts eating normally or dormant counterparts experiencing 9 to 10 hibernation cycles [14]. Gut microbial communities were more similar between ground squirrels shortly after rousing from hibernation (3 d after hibernation without eating) and in bouts of torpor and interbout arousal than to those in summer [15]. Moreover, the concentration of total SCFAs was significantly lower in ground squirrels shortly after rousing from hibernation than in those in summer [15]. The seasonality in gut microbiota found in *L. reevesii* hibernating (and thus, fasting) from late November to early March provides an inference that long-term fasting during hibernation significantly affects the composition of gut microbiota.

Numerous factors, including diet and temperature, affect the complexity of microbial co-occurrence networks [68,69]. An earlier study on the Brandt’s vole *Lasiopodomys brandtii* shows that a more complex co-occurrence network of gut microbiota may provide a more flexible microbial metabolic network [68]. As in *O. curzoniae* [8], co-occurrence networks of the gut microbial community were more complex in summer than in winter in both lizard species studied herein. To obtain more adequate energy from the diet, Brandt’s voles feeding *Cleistogenes squarrosa* rich in digestible nutrients have more diverse microbial communities and a complex network than those feeding *Stipa krylovii* poor in digestible nutrients [68]. The pattern of seasonality in the co-occurrence network complexity was similar between *E. multifasciata* and *L. reevesii*, whereas the direction of seasonal changes in negatively or positively correlated microbial taxa differed between the two species. Compared with conspecific lizards in summer, we found more positively correlated microbial taxa in co-occurrence networks in winter *L. reevesii* and more negatively correlated microbial taxa in winter *E. multifasciata*. Mutualistic or commensal taxa in microbial communities are positively correlated, and competitive taxa are negatively correlated [70]. The gut microbial co-occurrence network was dominant by positively correlated taxa in winter *L. reevesii*, presumably because long-term fasting during hibernation resulted in resource scarcity and put gut microbiota under high stress. This conforms to the stress gradient hypothesis, which suggests that more positively correlated taxa occur in the microbial co-occurrence network in high-stress environments (e.g., low-level resources and extreme temperatures) [68,71,72,73]. As environmental stress gradually intensifies, the competitive taxa involved in interspecific antagonism are gradually replaced by slow-growing, high-tolerant taxa, or by taxa providing direct benefits to other taxa [74,75]. Under benign environmental conditions, more negatively correlated taxa can enhance the stability of gut microbiota, because the competition between microbial taxa can reduce or minimize the resonance (co-oscillation) effect of environmental interference on microbial communities [76,77,78]. Thus, more negatively correlated taxa explain why the diversity and composition of gut microbiota are seasonally less variable in *E. multifasciata*.

## 5. Conclusions

Our study provides evidence that host species, season, and their interaction are significant sources of variation in the gut microbial diversity and composition in warm-climate lizards, allowing us to draw the following conclusions: First, seasonality in gut microbial alpha and beta diversities is evident in *L. reevesii* but not in *E. multifasciata*. Second, the Firmicutes/Bacteroidetes ratio significantly decreases and the relative abundance of Verrucomicrobia significantly increases in winter *L. reevesii*, helping the species adapt to long-term fasting during hibernation because bacteria of the phyla Bacteroidetes and Verrucomicrobia can use host-derived mucin glycans in the absence of dietary substrates. Third, the gut microbial community of summer *L. reevesii* is primarily dominated by Firmicutes (e.g., Lachnospiraceae) that can decompose plant fibers, and this finding adds evidence for dietary adaptation in omnivorous lizards. Taken together, our results allow the conclusion that gut microbial communities are seasonally more variable in warm-climate lizards hibernating in the winter months. Future work could usefully investigate other lineages of lizards to determine whether our conclusion is generalizable to all warm-climate species and can be used to guide conservation efforts for *L. reevesii*, an endangered lizard species on the list of the Class II Key Protected Wild Animals in China.

## Figures and Tables

**Table 1:** Descriptive statistics, expressed as mean ± SE (range), for alpha diversity indexes of gut microbiota in summer (S) and winter (W) in two host species of warm-climate lizards, *E. multifasciata* (E) and *L. reevesii* (L). *F* values of two-way ANOVA with host species and season as the fixed factors and significance levels are given in the table.

	Observed ASVs		Faith’s PD		Shannon		Evenness	
	Summer	Winter	Summer	Winter	Summer	Winter	Summer	Winter
*E. multifasciata*	395.6 ± 28.2 (272–496)	353.0 ± 25.5 (273–515)	28.0 ± 1.2 (23.3–33.2)	26.6 ± 1.2 (21.6–33.6)	6.2 ± 0.3 (5.0–7.1)	5.9 ± 0.2 (4.8–7.1)	0.72 ± 0.02 (0.60–0.79)	0.70 ± 0.02 (0.57–0.79)
*L. reevesii*	316.1 ± 16.1 (230–426)	235.6 ± 22.9 (142–348)	28.5 ± 1.1 (23.2–36.4)	23.8 ± 1.7 (17.0–32.5)	5.5 ± 0.2 (3.8–6.4)	4.0 ± 0.4 (1.9–5.8)	0.67 ± 0.02 (0.48–0.75)	0.50 ± 0.04 (0.26–0.69)
Host species (H)	*F*_1, 35_ = 18.04, *p* < 0.01; E > L		*F*_1, 35_ = 0.74, *p* = 0.40		*F*_1, 35_ = 21.51 *p* < 0.01; E > L		*F*_1, 35_ = 18.46, *p* < 0.01; E > L	
Season (S)	*F*_1, 35_ = 7.05, *p* = 0.01; S > W		*F*_1, 35_ = 4.98, *p* = 0.03; S > W		*F*_1, 35_ = 10.98, *p* < 0.01; S > W		*F*_1, 35_ = 10.24, *p* < 0.01; S > W	
H × S interaction	*F*_1, 35_ = 0.67, *p* = 0.42		*F*_1, 35_ = 1.42, *p* = 0.24		*F*_1, 35_ = 5.09, *p* = 0.03		*F*_1, 35_ = 5.99, *p* = 0.02	

## Data Availability

The raw sequence reads are available in the National Center for Biotechnology Information Sequence Read Archive (https://ncbi.nlm.nih.gov/sra, accessed on 20 May 2024) under BioProject ID PRJNA1113960.

## Supplementary Materials

The following supporting information can be downloaded at: https://www.mdpi.com/article/10.3390/microorganisms12101974/s1, Figure S1: Rarefaction curves based on observed ASVs (A) and Shannon index (B) for each sample; Figure S2: Results of principal coordinates analysis (PCoA) on gut microbiota based on Jaccard and Bray–Curtis distances; Table S1: Results of the permutational multivariate analysis of variance (PERMANOVA) on gut microbiota based on Jaccard, Bray–Curtis, unweighted UniFrac, and weighted UniFrac distances in two host species of warm-climate lizards, *E. multifasciata* and *L. reevesii*; Table S2: Comparison of relative abundance of ASVs between lizards in summer and winter.
