# Supplementary material for: Gut Microbial Communities Are Seasonally Variable in Warm-Climate Lizards Hibernating in the Winter Months
Source: Microorganisms. 2024 Sep 29;12(10):1974. doi: 10.3390/microorganisms12101974 (PMC11509526; doi:10.3390/microorganisms12101974)
Supplement: Supplementary file 1 [file microorganisms-12-01974-s001.zip › TableS1.pdf]

Table S1 Results of the permutational multivariate analysis of variance (PERMANOVA) on gut microbiota based on Jaccard, Bray-Curtis, unweighted UniFrac, and weighted UniFrac distances in two host species of warm-climate lizards, *E. multifasciata* and *L. reevesii*.

Variable	Jaccard			Bray-Curtis			unweighted UniFrac			weighted UniFrac		
	R^2	F	P	R^2	F	P	R^2	F	P	R^2	F	P
1) two species												
Host species	0.225	11.457	< 0.001	0.170	8.190	< 0.001	0.304	17.641	< 0.001	0.195	10.429	< 0.001
Season	0.046	2.362	< 0.01	0.052	2.485	< 0.01	0.053	3.054	< 0.01	0.067	3.573	< 0.01
Host species × Season	0.041	2.070	= 0.010	0.051	2.447	< 0.01	0.039	2.283	= 0.033	0.084	4.512	< 0.001
2) *E. multifasciata*												
Season	0.078	1.278	= 0.054	0.087	1.432	= 0.057	0.072	1.167	= 0.234	0.110	1.847	= 0.083
3) *L. reevesii*												
Season	0.130	2.998	< 0.001	0.143	3.349	< 0.001	0.158	3.762	< 0.001	0.221	5.687	< 0.001
