# Supplementary material for: Gut Microbial Communities Are Seasonally Variable in Warm-Climate Lizards Hibernating in the Winter Months
Source: Microorganisms. 2024 Sep 29;12(10):1974. doi: 10.3390/microorganisms12101974 (PMC11509526; doi:10.3390/microorganisms12101974)
Supplement: Supplementary file 1 [file microorganisms-12-01974-s001.zip › TableS2.pdf]

Table S2 Comparison of relative abundance of ASVs between lizards in summer and winter.

ASV ID	Taxonomy		Relative abundance %		logFC	adj *P*
	Family	Phylum	Summer	Winter		
E. multifasciata						
ASV101	Bacteroidaceae	Bacteroidetes	0.005 ± 0.005	0.085 ± 0.038	2.24	0.04
ASV533	Bacteroidaceae	Bacteroidetes	0.103 ± 0.045	–	–2.64	0.01
ASV760	Enterobacteriaceae	Proteobacteria	0.087 ± 0.030	–	–2.07	0.04
ASV955	Enterobacteriaceae	Proteobacteria	0.213 ± 0.094	–	–2.57	0.04
ASV1152	Enterobacteriaceae	Proteobacteria	0.077 ± 0.032	–	–2.20	0.04
ASV1184	Bacillaceae	Firmicutes	–	0.014 ± 0.005	1.39	0.02
ASV1367	Lachnospiraceae	Firmicutes	0.024 ± 0.008	0.001 ± 0.001	–1.55	0.04
L. reevesii						
ASV65	Ruminococcaceae	Firmicutes	0.082 ± 0.019	0.009 ± 0.006	–2.46	< 0.01
ASV108	Bacteroidaceae	Bacteroidetes	1.642 ± 0.658	–	–4.20	< 0.01
ASV165	[Odoribacteraceae]	Bacteroidetes	0.191 ± 0.092	–	–2.60	< 0.01
ASV348	Ruminococcaceae	Firmicutes	0.040 ± 0.020	1.205 ± 0.341	3.42	< 0.01
ASV562	Bacteroidaceae	Bacteroidetes	–	0.798 ± 0.565	3.03	< 0.01
ASV780	Lachnospiraceae	Firmicutes	–	0.063 ± 0.031	2.00	< 0.01
ASV923	Lachnospiraceae	Firmicutes	1.619 ± 0.412	0.115 ± 0.08	–2.95	< 0.01
ASV994	Bacteroidaceae	Bacteroidetes	–	0.039 ± 0.016	1.63	0.01
ASV1041	Lachnospiraceae	Firmicutes	2.273 ± 1.614	0.033 ± 0.011	–2.90	0.01
ASV1083	Ruminococcaceae	Firmicutes	0.054 ± 0.016	0.002 ± 0.002	–1.93	< 0.01
ASV1377	Ruminococcaceae	Firmicutes	0.110 ± 0.048	< 0.001	–2.23	< 0.01
ASV1410	Ruminococcaceae	Firmicutes	0.279 ± 0.091	–	–3.46	< 0.01
ASV1439	Rikenellaceae	Bacteroidetes	0.402 ± 0.304	–	–2.64	< 0.01
ASV1451	Verrucomicrobiaceae	Verrucomicrobia	2.018 ± 1.482	22.270 ± 8.435	4.75	< 0.01
ASV1550	Porphyromonadaceae	Bacteroidetes	0.246 ± 0.096	–	–2.63	0.01
ASV1691	Lachnospiraceae	Firmicutes	0.154 ± 0.044	0.010 ± 0.010	-2.58	< 0.01
ASV1704	Peptostreptococcaceae	Firmicutes	–	0.191 ± 0.117	2.38	< 0.01
ASV1717	Ruminococcaceae	Firmicutes	0.049 ± 0.013	–	–1.95	< 0.01
ASV1739	Clostridiaceae	Firmicutes	1.120 ± 0.699	–	–3.27	< 0.01
ASV1780	Christensenellaceae	Firmicutes	0.236 ± 0.060	0.002 ± 0.002	–3.60	< 0.01
ASV1800	Lachnospiraceae	Firmicutes	1.525 ± 1.098	0.001 ± 0.001	–3.64	< 0.01
ASV1809	Ruminococcaceae	Firmicutes	0.024 ± 0.007	–	–1.48	0.01
ASV1875	Rikenellaceae	Bacteroidetes	1.264 ± 0.472	–	–3.47	< 0.01
ASV1946	Lachnospiraceae	Firmicutes	0.052 ± 0.019	0.002 ± 0.002	–1.66	0.01
ASV1984	unclassified_o_Clostridiales	Firmicutes	0.038 ± 0.011	–	–1.99	< 0.01
ASV2053	Ruminococcaceae	Firmicutes	0.141 ± 0.036	0.008 ± 0.006	–2.88	< 0.01
ASV2066	Rikenellaceae	Bacteroidetes	0.229 ± 0.121	–	–2.67	< 0.01
ASV2074	Ruminococcaceae	Firmicutes	0.082 ± 0.019	0.009 ± 0.006	1.84	< 0.01
