# Supplementary figures and images for: Gut Microbial Communities Are Seasonally Variable in Warm-Climate Lizards Hibernating in the Winter Months
Source: Microorganisms. 2024 Sep 29;12(10):1974. doi: 10.3390/microorganisms12101974 (PMC11509526; doi:10.3390/microorganisms12101974)

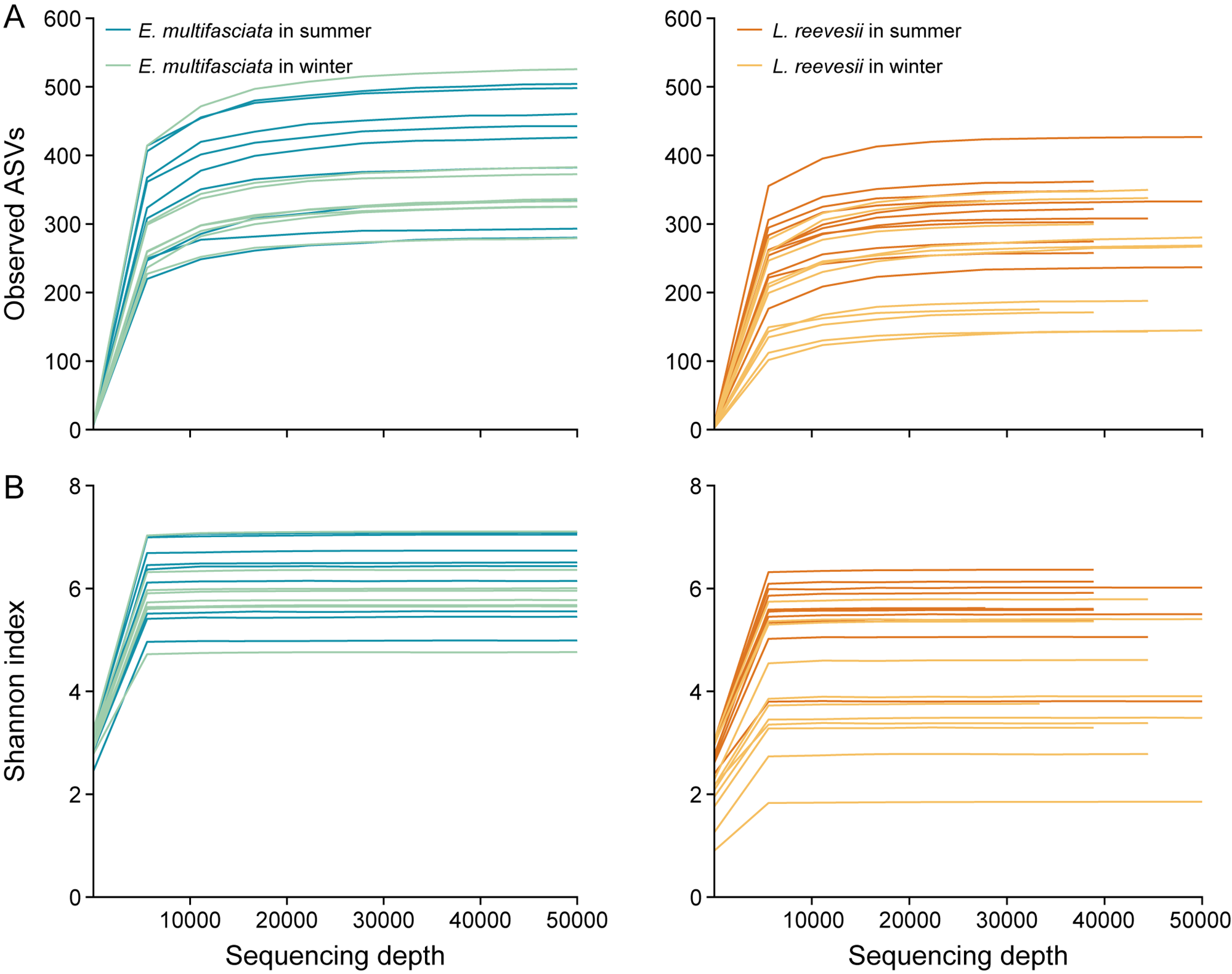

Supplement: Supplementary file 1 [file microorganisms-12-01974-s001.zip › FigureS1.tif]

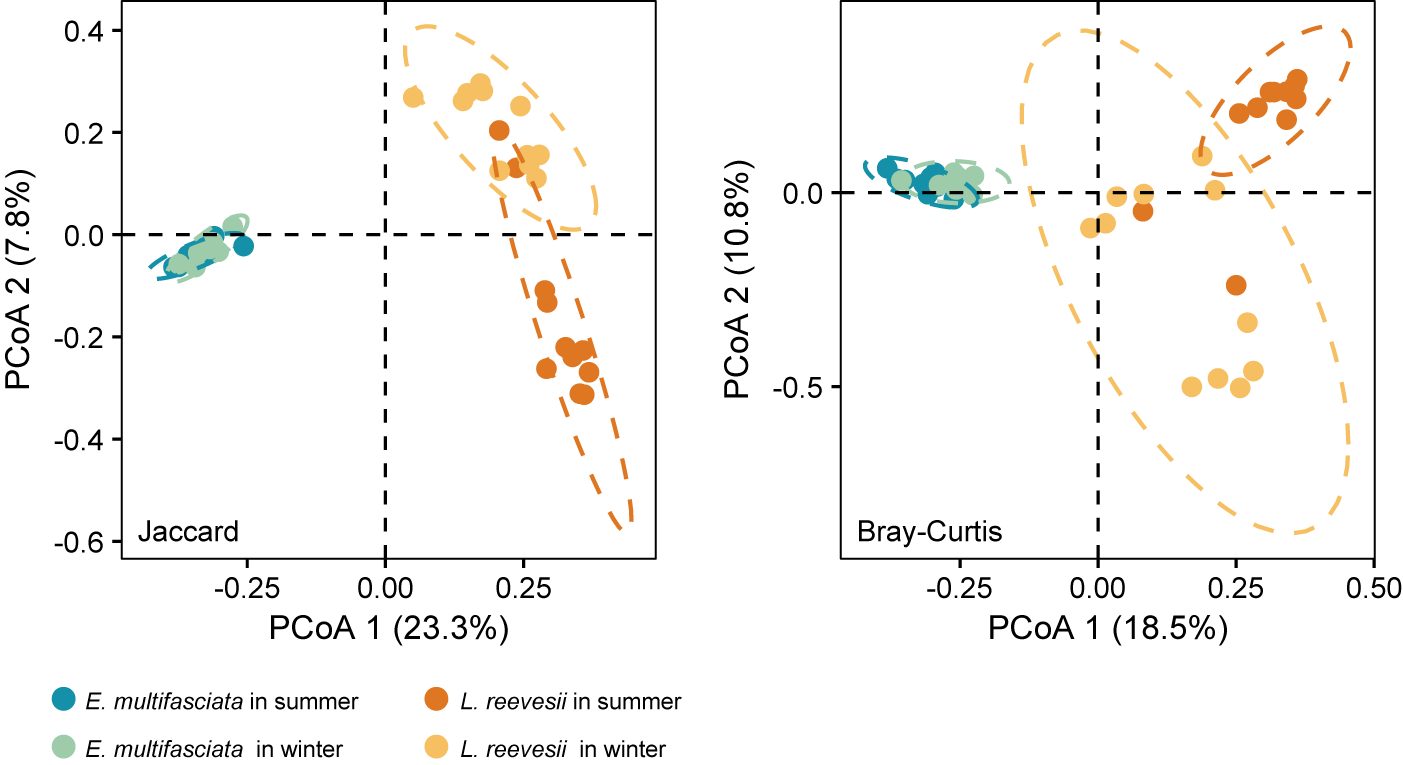

Supplement: Supplementary file 1 [file microorganisms-12-01974-s001.zip › FigureS2.tif]
